# Artificial spawning substrates and participatory research to foster cuttlefish stock recovery: A pilot study in the Adriatic Sea

**DOI:** 10.1371/journal.pone.0205877

**Published:** 2018-10-30

**Authors:** Fabio Grati, Gianna Fabi, Giuseppe Scarcella, Stefano Guicciardi, Pierluigi Penna, Martina Scanu, Simone Leoni, Francesco Riminucci, Cristina Frittelloni, Laura Gagliardini, Luca Bolognini

**Affiliations:** 1 Istituto per le Risorse Biologiche e le Biotecnologie Marine (IRBIM) – CNR, Ancona, Italy; 2 Istituto di Scienze Marine (ISMAR) – CNR, Venezia, Italy; 3 Istituto di Scienze Marine (ISMAR) – CNR, Bologna, Italy; 4 Agenzia Servizi Settore Agroalimentare delle Marche, Osimo Stazione, Italy; 5 Regione Marche, P.F. Caccia e Pesca, Ancona, Italy; University of Minnesota, UNITED STATES

## Abstract

This two-year study evaluates the effects of new management strategies directed at helping the recovery of Adriatic cuttlefish populations. The ability of three specially developed artificial spawning devices–seagrass collectors (SC; deployed on artificial reefs), longline collectors (LC; deployed at mussel farms), and trap collectors (TC; delivered to 19 professional and 54 recreational trap fishermen together with a dedicated logbook)–to attract egg deposition was tested. All devices were provided with a polyethylene floating rope 8 mm in diameter that served as a collector for egg deposition. Total rope length was 1,440 m in SC (2,880 segments of 0.5 m), 250 m in LC (500 segments of 0.5 m), and 250 m in TC (10 m per trap). Although the sites where SC and LC were deployed were sheltered from the action of destructive fishing gears, heavy winter storms destroyed the SC after a year. Most recreational fishermen and none of the professional fishermen provided detailed information on percent egg coverage on their collectors. The collectors attached to the three devices proved highly suitable for cuttlefish spawning, collecting more than 500,000 eggs on 2,440 m of rope. The analysis of egg diameter distribution suggested three laying events during the spawning season. The logbook data showed that egg number peaked in June. The present approach, combining habitat reconstruction and participatory research, has the potential to contribute to the recovery of cuttlefish stocks in the framework of a broader management plan.

## Introduction

The common cuttlefish *Sepia officinalis* (Linnaeus, 1758) is native to the Mediterranean Sea and the Eastern Atlantic, but inhabits a very wide range spanning from the Baltic and the North Sea to the waters around South Africa. It is a demersal species that is especially abundant in coastal waters on muddy and sandy bottoms covered with seaweed and phanerogams, but it can also be found down to a depth of ca. 200 m [1,2]. From 1990 to 2016, its total capture production from commercial fleets ranged from 10,470 tons in 1995 to 29,589 tons in 2012 [3]. *S*. *officinalis* is an important commercial resource in the Mediterranean Sea and especially in the Adriatic, where it is intensively exploited with bottom otter trawls, “rapido” (beam) trawls, trammel nets, fyke nets, and traps depending on its spatial and temporal distribution patterns [4,5]. It is also an important target species of recreational fisheries, which use traps and squid jigs [6]. According to a recent Italian Agriculture Ministry census, ca. 650,000 recreational and sport fishermen operate in the Adriatic Sea (https://www.politicheagricole.it); however, even though recreational fishing is governed by national regulations, its catches are not subject to reporting and are thus not included in national data collection and stock assessment programmes [7].

In the Adriatic Sea, common cuttlefish is exploited throughout the year by different fishing strategies and techniques. In early spring, adult cuttlefish concentrate in coastal spawning grounds, where they are targeted by small-scale and recreational fisheries [8]. In autumn, juveniles migrate to deeper offshore waters, where they are caught by bottom trawlers, either as a target species or as bycatch of vessels targeting demersal finfish, until late winter [5].

In the past few years, survey data (MEDITS and SOLEMON) collected in the Adriatic Sea have demonstrated significantly reduced *S*. *officinalis* abundance and biomass indices as well as spatial distribution [9, 10]. According to a surplus production model applied in the framework of the General Fisheries Commission for the Mediterranean (GFCM) Working Group for Demersal Species, the current stock biomass is below the safe biological limit (B_MSY_; [10]). The situation may be a direct result of the removal of adults and juveniles by fishing activities, the consequence of their interference with breeding events, and/or an indirect effect of the destruction of essential habitats and of resultant changes in trophic webs and community structures [11]. *S*. *officinalis* has a lifespan of about two years; since the spawning season is followed by mass adult mortality, marine environmental conditions are held to exert a strong effect on recruitment and spatial distribution [12]. Moreover, since egg masses are attached in clusters to seagrass, tube worms, drowned trees, ropes, and traps [13], recruitment strongly depends on the availability of spawning substrates. This may be a critical factor, especially in Adriatic coastal areas, where habitat loss and degradation are particularly severe due to human activities [11]. The choice of the spawning substrate has been reported to depend on the female’s preferences, which seem to favour tubular surfaces less than 10 mm in diameter [14]. Since cuttlefish eggs take from 20 to 50 days to open [15], ropes and traps are often completely covered with eggs throughout the spawning season [16]. Although egg removal and destruction from set gears is banned by a number of local regulations, in Italy removal with highly destructive devices (e.g. pressure washers) to ensure gear efficiency is quite frequent [11, 16, 17]. All the above factors, combined with natural predation [18, 19], are likely to be involved in the decline of Adriatic cuttlefish stocks [9].

To date, management measures such as trawling bans within 3 nm of the coast, minimum codend mesh size, and trawling closures have failed to prevent stock reduction or to foster stock recovery. Given the urgent need for action, a different approach may be warranted. A participatory research process involving a direct, active role for stakeholders, especially artisanal and recreational fishermen, has the potential to raise their awareness of the natural resources they exploit and to help identify solutions that can improve the success of management actions [11, 20].

Based on these considerations, a polyethylene floating rope was mounted on three specially developed devices to attract cuttlefish egg deposition [21]. The three types of devices–seagrass collectors (SC; [22]), longline collectors (LC; [23]), and trap collectors (TC; [24])–were designed so as to be easy to handle and to require no maintenance during their use at sea [16]. They were tested in the field in collaboration with artificial reef managers, professional mussel farmers, and recreational and professional trap fishermen. The study was conducted in the western Adriatic Sea to test the ability of this new management strategy to foster cuttlefish stock recovery and to induce active stakeholder involvement.

## Material and methods

### Ethics statement

The research vessel R/V Tecnopesca II (ISMAR-CNR, Ancona, Italy) was used for the study. All eggs were collected during common survey procedures without any additional experimental catches. The study was not subject to Health Ministry authorisation because it involved no animal experiments, and hatching experiments do not cause pain, suffering, distress, or lasting harm (Italian decree no.116/92 and European Directive 2010/63/EU). Fishermen participated on a voluntary basis and gave their verbal consent to the use of the information collected through their logbooks. No specific permissions were required for sampling at sea and the field studies did not involve endangered or protected species.

### Study area

**Fig 1:**
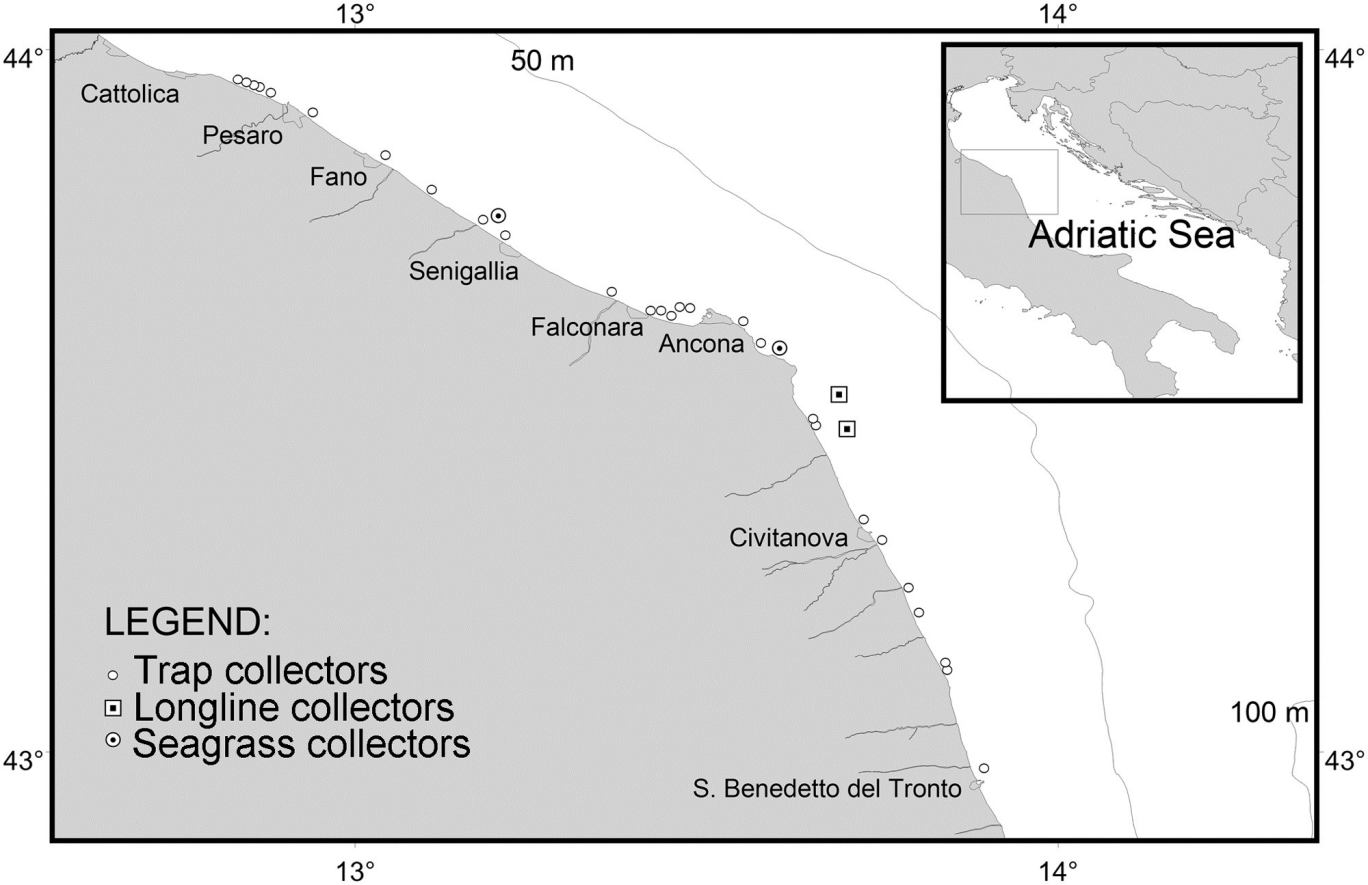
Study area. Image of the study area showing the position of the seagrass collectors, longline collectors, and trap collectors.

The study was carried out in the western Adriatic Sea along a stretch of coastline measuring about 170 km, from Cattolica to San Benedetto del Tronto (Fig 1), which is a narrow epicontinental basin characterised by a low topographic gradient (average ~ 0.02°). Salinity is low because the Adriatic receives about 1/3 of all the freshwater flowing into the Mediterranean, acting as a dilution basin. Water temperature in the coastal area usually ranges from 7 °C in winter to 28 °C in summer.

The area is characterised by eutrophic conditions and hosts several human activities, including aquaculture and recreational and professional fisheries [25]. Coastal fisheries include 421 small-scale vessels using set nets and traps, 221 hydraulic dredges for baby clam (*Chamelea gallina*), and 816 recreational fishermen using cuttlefish traps (pers. comm. Marche Regional Authority). Twenty-two longline mussel farms, growing *Mytilus galloprovincialis*, occupy a sea surface of 755,600 m^2^, producing about 3,000 tons of mussels each year. Human activities compete for space and resources: hydraulic dredges compete with set gears, small-scale fisheries with mussel farms, illegal trawling inside the 3 nm buffer from the coast competes with small-scale fishermen, and recreational fishermen compete with small-scale fishermen [26].

The area also hosts seven artificial reefs, 5 medium-sized or large ones (from 0.02 km^2^ to 0.80 km^2^) laid for coastal protection and finfish repopulation and 2 small reefs (900 m^2^ and 6,000 m^2^) set up for research purposes [27, 28].

### Artificial spawning substrates

Three types of devices designed to be deployed at artificial reefs, mussel farms, and in the open sea were developed for the study. All bore segments of polyethylene floating rope 8 mm in diameter.

#### Seagrass collectors

**Fig 2:**
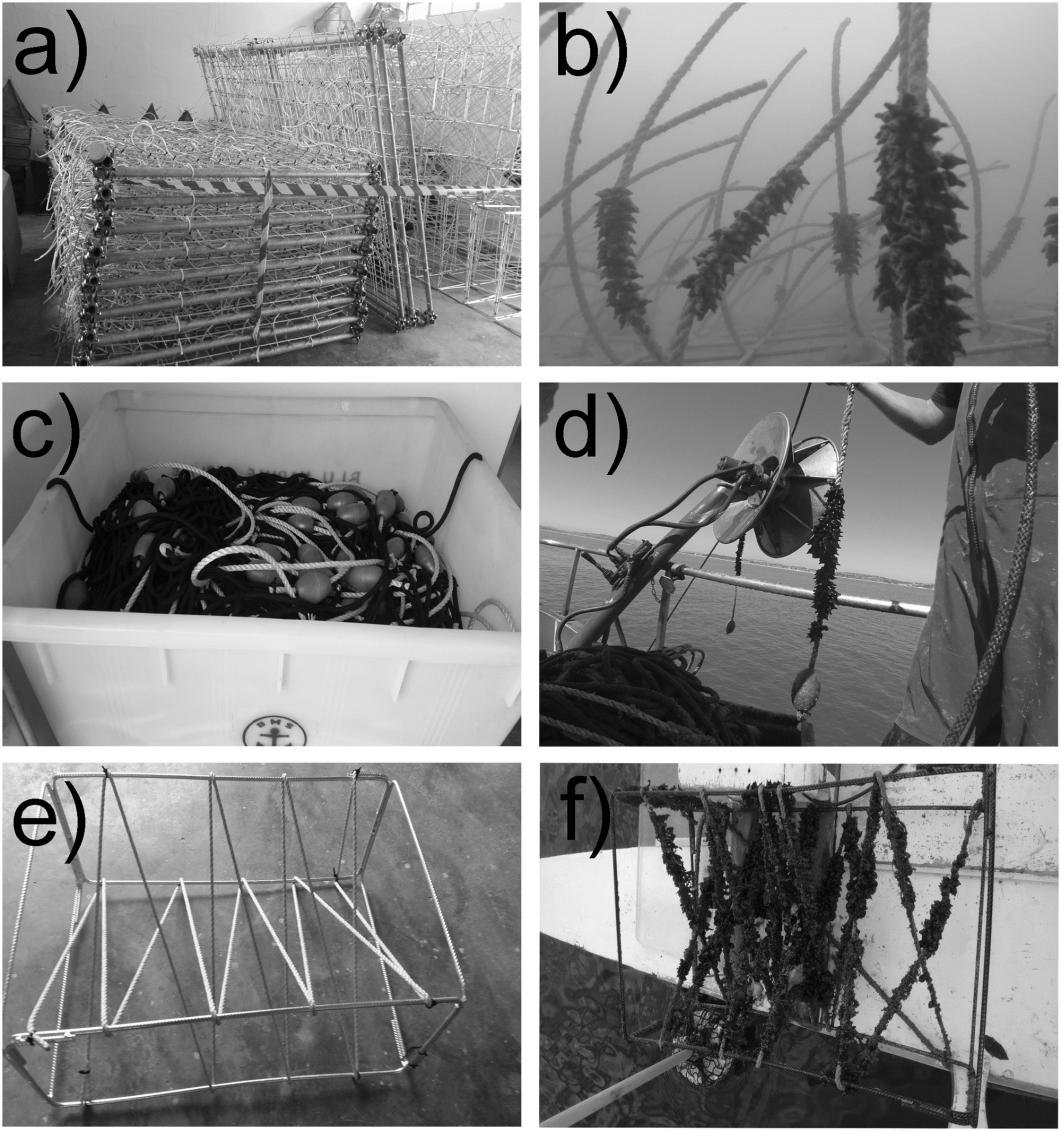
Egg collectors. a) Seagrass collectors; b) seagrass collector bearing cuttlefish eggs; c) longline collectors; d) longline collectors bearing cuttlefish eggs; e) trap collector; f) trap collector bearing cuttlefish eggs.

SC (n = 18) were made from electro-galvanized iron wire mesh (diameter 6 mm, 150 x 150 mm mesh, sheet size 2 x 3 m; Fig 2a and 2b). Each SC was endowed with 160 segments of polyethylene floating rope 50 cm in length and 8 mm in diameter, in line with a similar study [16]. In April 2015, nine SC were deployed in front of Senigallia (Fig 1), beside a small artificial reef that was set up for research purposes by CNR-ISMAR in 1987. The other nine SC were fixed to the seabed by scuba divers beside a medium-sized artificial reef laid by the local consortium of mussel farmers in Portonovo Bay (Fig 1) in 1990. They were placed close to the artificial reef for protection against heavy storms. At both sites, the 9 devices were linked together in a single set to increase their resistance to the waves.

#### Longline collector

LC (n = 4) were designed to be deployed at longline mussel farms. Each consisted of a 400 m long lead rope (200 grams per meter) with 250 collectors, i.e. 50 cm segments of polyethylene floating rope bearing a float of 40 g at the free end (Fig 2c and 2d). The LC were deployed at two mussel farms (2 LC each) located south of Ancona (Fig 1) in April 2015 and April 2016, at the beginning of the cuttlefish spawning period.

#### Trap collector

TC were designed to be used by professional and recreational fishermen in spring, during the spawning period. They consisted of a galvanized iron frame (80 x 50 x 30 cm) with the polyethylene floating rope (10 m) coiled around it (Fig 2e and 2f). The TC were similar to the traps commonly used by local fishermen except for the net, to allow cuttlefish to escape [29]. The TC were delivered to 73 local fishermen, 54 recreational (1 each) and 19 professional (3 each) on the occasion of ad hoc seminars where the CNR-ISMAR researchers illustrated the aims of the project and provided information on participation. Participants were given logbooks in which to record the data needed for the research. The TC were deployed in March (Fig 1), at the beginning of the spawning season, and recovered in July.

### Data collection

#### Seagrass collectors

During the cuttlefish spawning season (March-July 2015 and 2016), the SC were examined monthly by scuba divers for their conditions and to determine how many collectors bore eggs. A random sample of 10 collectors was retrieved by scuba divers 40 days (May 2015) and 80 days (July 2015) after deployment and taken to the laboratory for analysis. These collectors were not replaced. The number of eggs on each collector was counted; a sample of 100 eggs was randomly selected to measure their diameter with a vernier caliper down to the lowest mm. No SC were retrieved in 2016, probably because heavy storms destroyed the devices or buried them under the soft sediment.

#### Longline collectors

LC sampling was carried out in May 2015, July 2015, June 2016, and July 2016. LC were taken on board; all collectors bearing eggs were counted and removed to be analysed in the laboratory as described for the SC.

#### Trap collectors

Fishermen were asked to examine their TC each time they went fishing and to record in their logbook the presence of eggs, the percent coverage on each TC, and the starting and ending date of their fishing season. A drawing on the first page of the logbook illustrated an easy way to assess the egg percent coverage (< 25%; 25–50%; 50–75%; > 75%) on the collector.

### Data analysis

#### Seagrass collector

Since no SC were recovered in 2016, the mean number of eggs per collector on this substrate (±1 standard deviation) could be calculated only for the samples taken in 2015.

#### Longline collector

The mean number of eggs per collector (±1 standard deviation) and their diameter distribution were calculated for each sampling. For the analysis of egg diameter distribution each data group was fitted with a lognormal distribution *Lognormal*(*μ*, σ^2^). σThe mean $\bar{x}$ and the standard deviation σ on the original scale were calculated as $\bar{x}=e^{\left(\mu +\sigma^{2}\right)/2}$ and $\sigma =\sqrt{\left(e^{\sigma^{2}}-1\right)\;e^{2\mu +\sigma^{2}}}$. When the data showed a unimodal dispersion, a single lognormal distribution was fitted. If the histogram showed more than one peak, an equal number of lognormal distributions was considered; in such cases, the fitting curve was expressed as a sum of lognormal distributions *Lognormal*_*i*_(*μ*_*i*_, σ_*i*_^*2*^):

$$fitting\;curve=\sum_{i}f_{i}Lognormal\left(\mu_{i},\;\sigma_{i}{}^{2}\right)$$

where *f*_*i*_ is the fraction of the total data with the constrain ∑_*i*_*f*_*i*_ = 1. The fitting was carried out using the empirical distribution function [30], to overcome the histogram bin width. In short, the diameter data of each data group were sorted from the lowest to the highest; to each ranked datum *i*, a corresponding estimate of the empirical distribution probability *P*_*i*_ was attributed according to:

$$P_{i}=\frac{i}{n}$$

where *i* is the datum index and *n* is the total data number. The main statistical parameters of the distribution, *μ*_*i*_, σ_*i*_^*2*^ and the relative data fraction *f*_*i*_, were then estimated by nonlinear fitting of the data set {x_i_, P_i_} with a lognormal distribution *Lognormal*(*μ*, σ^*2*^), or with a sum of lognormal distributions if multiple peaks were evident. The nonlinear fit was performed using the commercial math software (Mathematica version 11.1, Wolfram Research Inc. Champaign, IL, 2017). Since the two mussel farms were relatively close to each other, their data were pooled.

#### Trap collector

The number of eggs attached on the TC was estimated on the basis of the median percent egg coverage value reported by the fishermen in their logbook, which was > 75% (300 eggs per 0.5 m; median of values ranging from 200 to 485 eggs/0.5 m). Extrapolation of this value to the length of each segment (10 m) indicated that a coverage < 25% corresponded to 1,500 eggs, one of 25–50% corresponded to 3,000 eggs, a coverage of 50–75% corresponded to 4,500 eggs, and a coverage > 75% corresponded to 6,000 eggs. The total number of eggs deposited on the TC from March to July 2015 and 2016 was estimated for the study.

## Results

### Seagrass collectors

In May 2015, 290 (20%) of the 1,440 collectors deployed by the artificial reef at Portonovo bore cuttlefish eggs. The mean number of eggs per collector was 78.3 ± 12.8 and their mean diameter was 6.1 ± 0.7 mm. As regards the SC deployed at the Senigallia artificial reef, only one (0.07%) of the 1,440 collectors bore eggs. There were 53 eggs with a mean diameter of 8.3 ± 0.7 mm. In July 2015, 565 (39.2%) collectors deployed at the Portonovo site bore eggs; their mean number per collector was 144.3 ± 144.2 and their mean diameter was 7.7 ± 2.7 mm, whereas none of those collectors deployed by the Senigallia artificial reef bore eggs.

### Longline collectors

**Fig 3:**
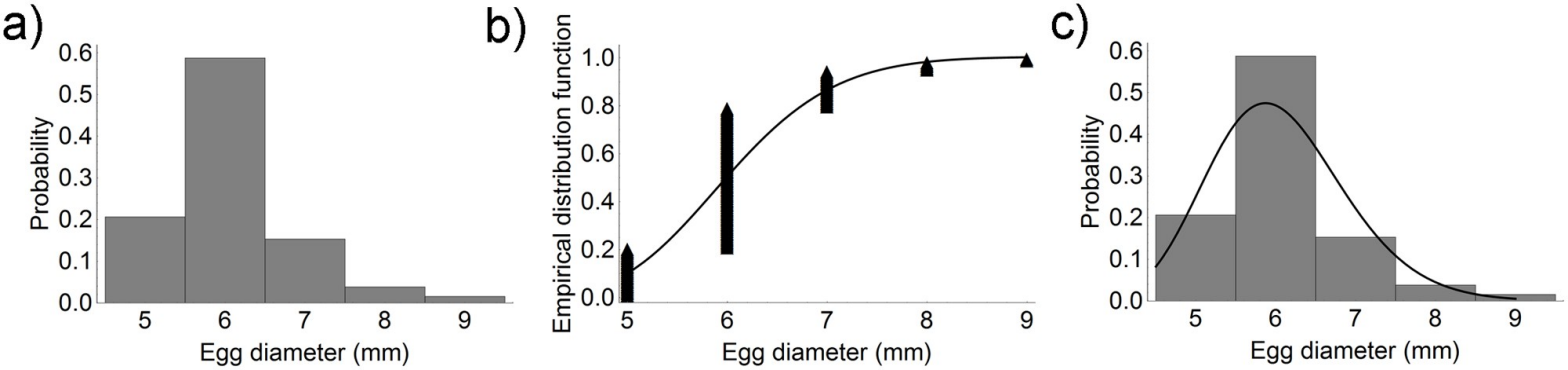
Egg diameter distribution in May 2015. a) Egg diameter distribution on longline collectors. b) Experimental data and fitted lognormal distribution. c) Comparison between the histogram and the probability density function of the fitted lognormal curve.

In May 2015, cuttlefish eggs were detected on 90 (36%) collectors; mean egg number per collector was 152.2 ± 119.45 and mean egg diameter was 6.1 ± 0.8 mm. Since the egg diameter distribution showed a single peak at 6 mm, only one *Lognormal*(*μ*, *s*^*2*^) distribution was fitted to the experimental data (Fig 3).

**Fig 4:**
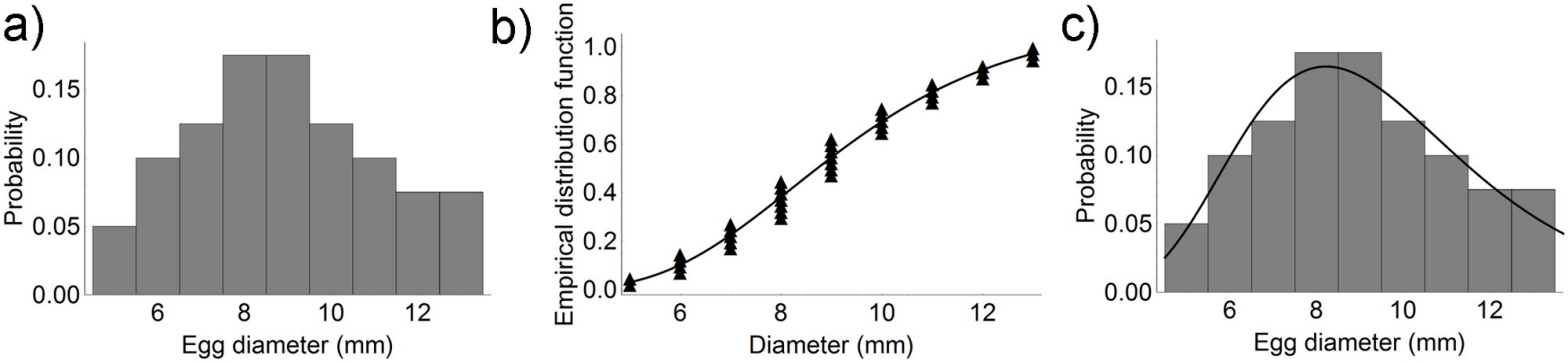
Egg diameter distribution in July 2015. a) Egg diameter distribution on longline collectors. b) Experimental data and fitted lognormal distribution. c) Comparison between the histogram and the probability density function of the fitted lognormal curve.

In July 2015, eggs were found on 220 (88%) collectors; their mean number per collector was 174.9 ± 120.2 and their mean diameter was 8.9 ± 2.2 mm. Their diameter distribution and fitting to the experimental data are reported in Fig 4. The probability distribution function (PDF) of the fitted lognormal curve superimposed on the experimental data clearly shows higher values compared with May 2015.

**Fig 5:**
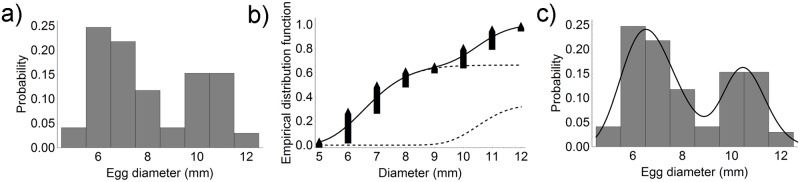
Egg diameter distribution in June 2016. a) Egg diameter distribution on longline collectors. b) Experimental data, the two fitted lognormal distributions (dashed line), and the overall fitting curve (solid line). c) Comparison between the histogram and the probability density function of the overall fitted curve.

In June 2016, 112 (45%) collectors bore cuttlefish eggs; their mean number per collector was 172.4 ± 146.7. Since the histogram of the diameter values clearly shows a bimodal distribution, with peaks at 6 mm and 9 mm, the sum of two lognormal distributions was fitted to the experimental data (Fig 5). The experimental data show a mixture of two distributions, a smaller distribution centred at a lower value, and a broader distribution. On the original scale, the lognormal distribution of the smaller population had a mean value $\overset{-}{x}$ and a standard deviation *s* of 6.8 mm and 1.1 mm, respectively, whereas the $\overset{-}{x}$ and *s* of the larger population were 10.6 mm and 0.8 mm, respectively. The smaller distribution accounted for ca. 66% of the total data.

**Fig 6:**
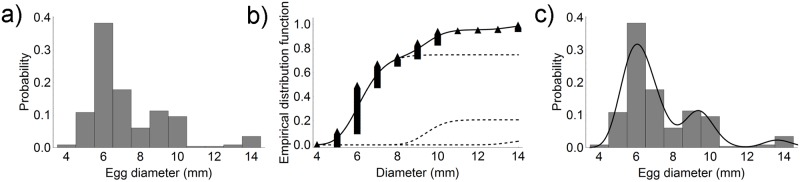
Egg diameter distribution in July 2016. a) Egg diameter distribution on longline collectors. b) Experimental data, the three fitted lognormal distributions (dashed line) and the overall fitting curve (solid line). c) Comparison between the histogram and the probability density function of the overall fitted curve.

In July 2016, cuttlefish eggs were detected on 200 (80%) collectors; the mean number per collector was 115.6 ± 103.1. The diameter distribution showed a mixture of three distributions with peaks at 6 mm, 9 mm, and 14 mm (Fig 6). Their $\overset{-}{x}$ and *s* were 6.3 ± 0.9 mm, 9.5 ± 0.8 mm, and 13.7 ± 0.8 mm, respectively. The smallest distribution accounted for 75% of the total data, the middle distribution for 21%, and the largest distribution for ca. 5%.

### Trap collectors

**Fig 7:**
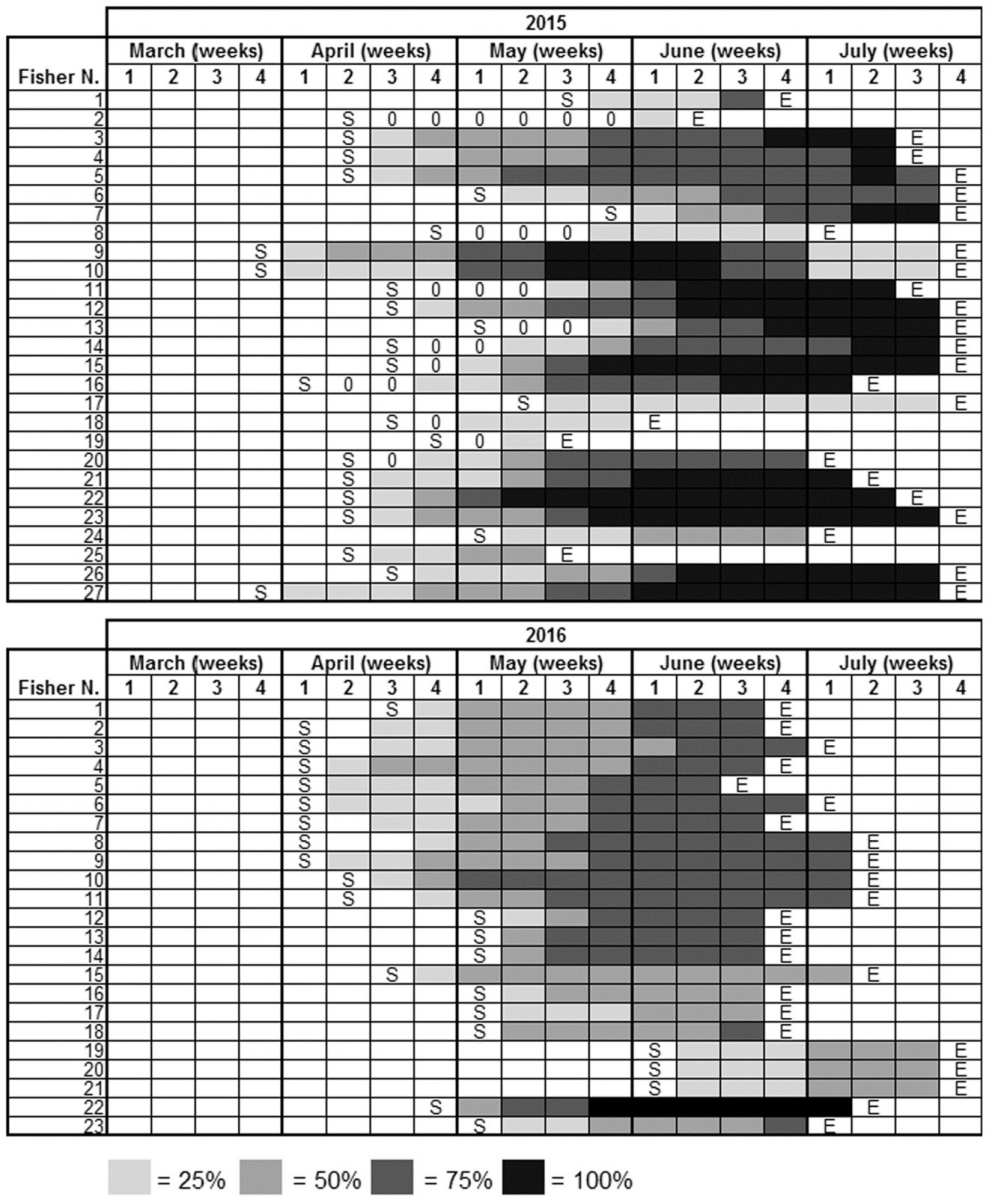
Egg percent coverage on trap collectors. Egg percent coverage recorded by recreational fishermen in 2015 and 2016. S = start of the fishing season; 0 = no eggs; E = end of the fishing season.

A total number of 27 recreational fishermen (50% of the recreational fishermen involved in the pilot study) recorded the percent egg coverage of their collectors during the 2015 fishing season (Fig 7) in their logbook. In 2016, 23 recreational fishermen returned the logbook. None of the 19 professional fishermen returned it. The TC were homogenously distributed along the study area (170 km of coastline), except for two hotspots around Pesaro and Ancona (Fig 1).

**Fig 8:**
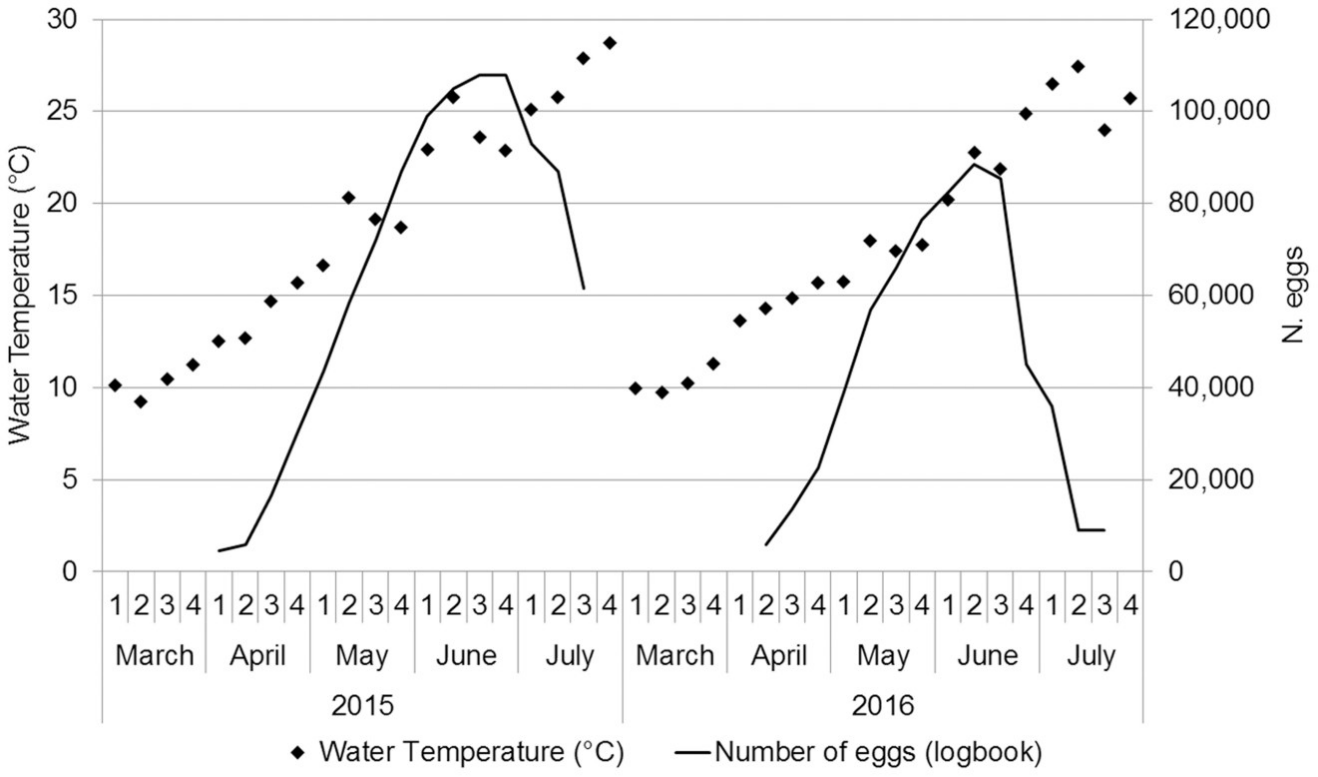
Water temperature and total number of eggs found on trap collectors. Water temperature (diamonds) and total number of eggs (continuous line) laid on the collectors deployed by recreational fishermen in 2015 and 2016 (logbook data).

In 2015, 17 (63%) TC were totally covered with cuttlefish eggs, and the peak of the spawning season lasted from mid-May to mid-July (Fig 7). In 2016, only one TC was completely covered, and the peak of the spawning season lasted less (Fig 7). In particular, egg deposition began in the first two weeks of April (water temperature, 12–14 °C; Fig 8) and their number peaked in the last two weeks of June 2015 (108,000 eggs) and the second week of June 2016 (88,500 eggs; Fig 8), when the water temperature reached 22–23 °C. In 2016 hatching was two weeks in advance compared with 2015.

## Discussion and conclusions

This is the first study assessing the effects of a new management strategy aimed at fostering the recovery of cuttlefish stocks in the Adriatic Sea. Besides providing useful data on this issue, the study also supplies novel information on the biology of this species in the study area. In particular, the LC logbook data showed that the 2015 spawning season was richer and more extended, with a single massive spawning compared with three distinct events in 2016. The latter findings are in line with those of Boletzky [12], who observed the release of fertilized eggs over a period of several weeks, with several spawning events. The frequency of these events may be the result of the combination of two factors: a single specimen spawning several times and/or successive arrival in the area of specimens with different sizes [31].

The study findings are also in line with the data collected in the framework of the SOLEMON trawl survey [10], which found that the cuttlefish abundance index in the central and northern Adriatic (GFCM Geographical SubArea 17) was greater in November 2014 (361.3 individuals/km^2^) than in November 2015 (252.9 individuals/km^2^). In fact, the numerous spawners seen in autumn 2014 migrated inshore four months later (March 2015), laying the large number of eggs that were recorded in the present study. Consequently, the small number of adults seen in autumn 2015 may be the reason for the poor spawning season of spring 2016.

**Fig 9:**
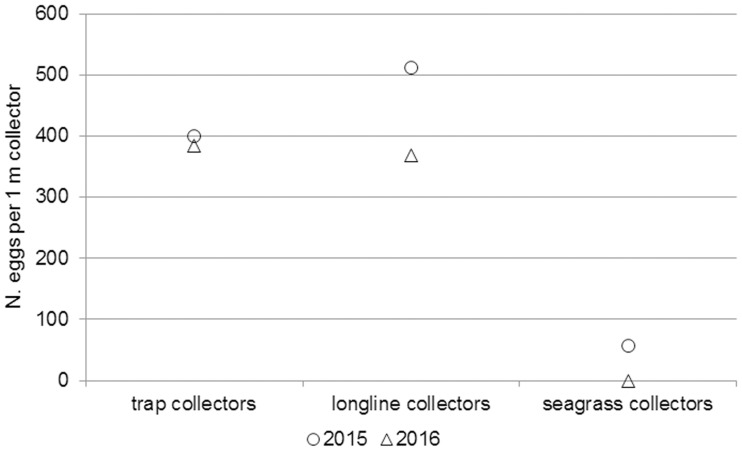
Number of eggs per meter of collector. Estimated number of eggs per meter laid on trap collectors, longline collectors and seagrass collectors in 2015 and 2016.

As regards the devices developed to foster egg survival during the spawning season, the study confirmed the high efficiency of polyethylene floating ropes (8 mm diameter) as cuttlefish egg collectors described by Blanc and Daguzan [16] and Boletzky [12], since all three types of devices proved to be similarly suitable. The smaller percentage of SC collectors bearing eggs can probably be explained by the fact that they lacked floats (and therefore could not remain in vertical position) and/or with their close proximity to each other. This is also confirmed by the different number of eggs per meter (Fig 9), i.e. 368 on LC, 512 on TC, and only 56 on SC (2015 data). Moreover, although the SC had been designed to remain permanently at sea, given the steep cost of deploying them each spawning season, they proved unsuitable. Their failure was mainly due to their tendency to sink in soft sediments–since at the Senigallia artificial reef, where the seabed is muddy, they were completely covered by sediment already a few months after deployment–as well as to their poor resistance to rough sea. Efforts to find technical solutions, especially to improve their resistance to adverse weather conditions in open sea areas, are clearly warranted, since the western Adriatic is affected by strong winds (i.e. bora) and storms [32].

The LC were designed to be easy to handle and recover at the end of the spawning period, to prevent damage in winter. These devices proved suitable for deployment in suspended mussel farms. However, they required washing to remove fouling and egg residues at the end of the spawning season (July-August); moreover, their need to be stored until the beginning of the spring (February/March) involves work that in the present case was performed by the CNR-ISMAR researchers.

As regards the TC, they were deployed by professional and recreational fishermen together with their own gear (traps and set nets in the former case and traps in the latter). As a result, protection against destructive gears and storms as well as logbook compilation were managed by the fishermen themselves, voluntarily and free of charge. The study findings indicate that 50% of the recreational fishermen involved in the project were both motivated and aware of the importance of actions undertaken to foster stock recovery. This was reflected in their logbooks, which provided useful, detailed information on percent egg coverage. In contrast, none of the 19 professional fishermen who volunteered for the project compiled the logbook.

**Fig 10:**
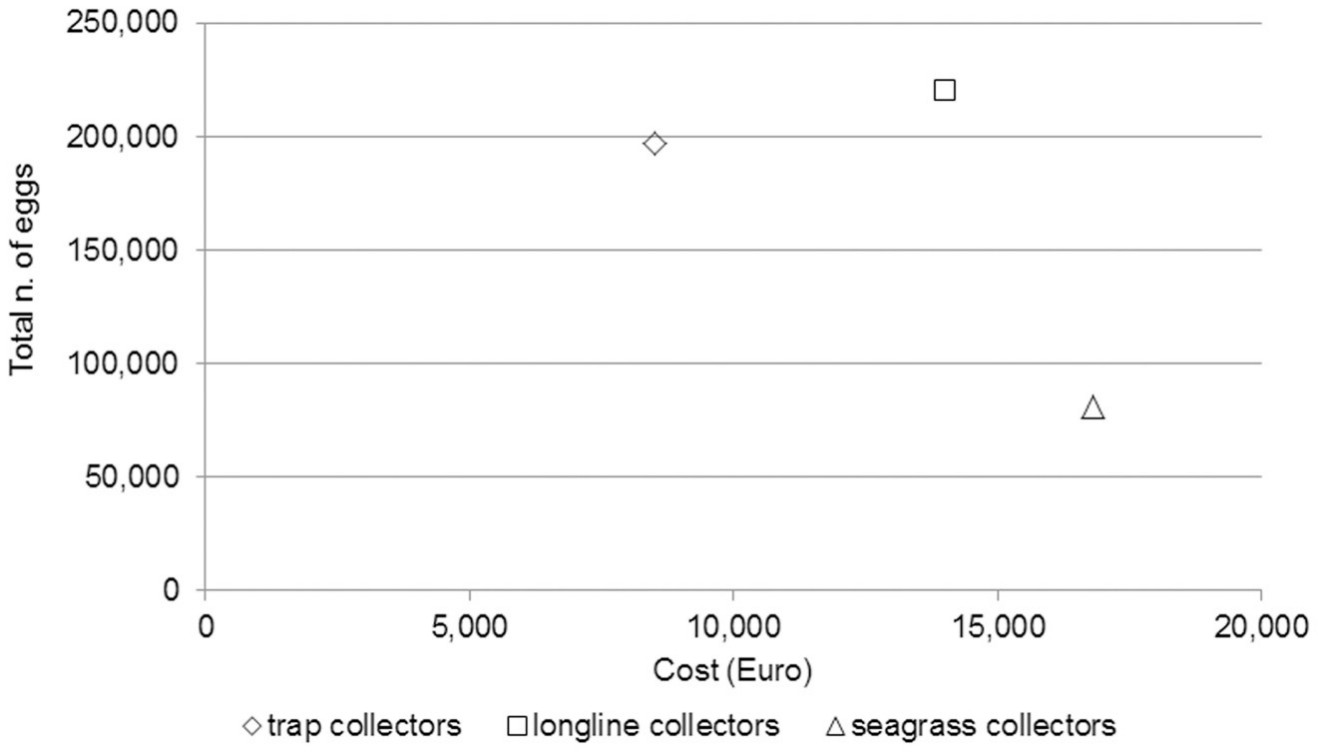
Cost-benefit analysis. Estimated number of eggs laid on trap, longline and seagrass collectors and cost of each device.

Overall, LC and TC both showed good efficiency and similar maintenance requirements; they were also much more cost-effective than SC (Fig 10). The large-scale adoption of longlines and/or traps would clearly require the cooperation of mussel farmers and fishermen, both professional and recreational; additional effort should thus be devoted to increase their participation in future activities.

The present results have prompted the Regional Authority of Marche to offer benefits to mussel farmers who will include LC in their concession application / renewal. Similar actions could be undertaken to achieve a greater cooperation by fishermen, especially professional ones, to strengthen participatory research. Interestingly, logbooks are widely used in the stock assessment programmes of the Canadian Department of Fisheries and Oceans (DFO) and the US National Marine Fisheries Service (NMFS). The recreational equivalent, angler diaries, are also used to assess stocks and the accuracy of recreational fishing surveys by several agencies in both countries [33]. Given the above considerations, the approach developed and tested in the present study can easily be replicated and extended to other areas and/or cuttlefish species, providing an important step towards the co-management of fishery resources.
